# The Role of Mitochondria in Drug-Induced Kidney Injury

**DOI:** 10.3389/fphys.2020.01079

**Published:** 2020-09-04

**Authors:** Zhibo Gai, Ting Gui, Gerd A. Kullak-Ublick, Yunlun Li, Michele Visentin

**Affiliations:** ^1^ Experimental Center, Shandong University of Traditional Chinese Medicine, Jinan, China; ^2^ Department of Clinical Pharmacology and Toxicology, University Hospital Zurich, University of Zurich, Zurich, Switzerland; ^3^ Mechanistic Safety, CMO & Patient Safety, Global Drug Development, Novartis Pharma, Basel, Switzerland; ^4^ Innovation Research Institute of Traditional Chinese Medicine, Shandong University of Traditional Chinese Medicine, Jinan, China; ^5^ The Third Department of Cardiovascular Diseases, Affiliated Hospital of Shandong University of Traditional Chinese Medicine, Jinan, China

**Keywords:** antibiotic, anticancer, antiviral, drug-induced kidney injury, mitochondria, oxidative stress, proximal tubular cells

## Abstract

The kidneys utilize roughly 10% of the body’s oxygen supply to produce the energy required for accomplishing their primary function: the regulation of body fluid composition through secreting, filtering, and reabsorbing metabolites and nutrients. To ensure an adequate ATP supply, the kidneys are particularly enriched in mitochondria, having the second highest mitochondrial content and thus oxygen consumption of our body. The bulk of the ATP generated in the kidneys is consumed to move solutes toward (reabsorption) or from (secretion) the peritubular capillaries through the concerted action of an array of ATP-binding cassette (ABC) pumps and transporters. ABC pumps function upon direct ATP hydrolysis. Transporters are driven by the ion electrochemical gradients and the membrane potential generated by the asymmetric transport of ions across the plasma membrane mediated by the ATPase pumps. Some of these transporters, namely the polyspecific organic anion transporters (OATs), the organic anion transporting polypeptides (OATPs), and the organic cation transporters (OCTs) are highly expressed on the proximal tubular cell membranes and happen to also transport drugs whose levels in the proximal tubular cells can rapidly rise, thereby damaging the mitochondria and resulting in cell death and kidney injury. Drug-induced kidney injury (DIKI) is a growing public health concern and a major cause of drug attrition in drug development and post-marketing approval. As part of the article collection “Mitochondria in Renal Health and Disease,” here, we provide a critical overview of the main molecular mechanisms underlying the mitochondrial damage caused by drugs inducing nephrotoxicity.

## Introduction

Kidney Disease: Improving Global Outcomes (KDIGO) guidelines define acute kidney injury in patients with normal renal function (serum creatinine of 1.3–1.5 mg/dl) as: (i) a serum creatinine increases by 0.3 mg/dl or more within 48 h, (ii) a serum creatinine increases by 50% or more from the baseline within the previous 7 days, and/or (iii) a urine volume is lower than 0.5 ml/kg/h for 6 h (Palevsky et al., 2013). It has been estimated that 14–26% of all acute kidney injury cases are induced by drugs. Drug-induced kidney injury (DIKI) not only represents a concerning public health issue but also substantially contributes to the rate of drug attrition in drug development and post-marketing approval (Mehta et al., 2004; Uchino et al., 2005; van der Meer et al., 2010; Moffett and Goldstein, 2011; Hoste et al., 2015). The incidence of DIKI seems to rise when we consider new prescription drugs, likely because (i) some adverse drug reactions can only be observed in large, genetically heterogeneous populations, only reachable several years upon marketing authorization, (ii) recognition of cases of DIKI is particularly challenging for medications not previously associated with kidney damage, because there is no consensus on the definition of DIKI, and (iii) some regulatory agencies have nearly halved the approval review times in the last 20 years, even in the absence of significant advantages for patients in comparison to existing medications (Olson, 2004; van der Meer et al., 2010; Awdishu and Mehta, 2017).

The functional unit of the kidney is the nephron, which is composed of a glomerulus and a tubule. The glomerulus filters the blood, retaining cells and large proteins, producing an ultrafiltrate mainly made of small molecules, from nutrients to ions. The ultrafiltrate enters the tubule, in which highly specialized cells at various segments (proximal tubule, Henle’s loop, and distal tubule) contribute to modify the nascent urine by removing substances from the tubular fluid (reabsorption) or adding substances to the tubular fluid (secretion). For a long time, the recognition of the primary site of the renal damage induced by drugs has been complicated because only cases associated with a change in serum creatinine, which is a sign of extended tissue damage to the point of renal dysfunction, were recognized. With the introduction in preclinical and clinical development of a panel of segment-specific markers such as neutrophil gelatinase-associated lipocalin (Ngal; Mishra et al., 2003) and urinary kidney injury molecule-1 (Kim-1; Ichimura et al., 1998; Han et al., 2002), thereby more explicitly addressing the etiology of the renal insult and identifying the injury before the serum creatinine elevates, it could be established that the majority of the DIKI cases have a tubular etiology with primary glomerular injury being relatively uncommon (Mehta et al., 2015). DIKI can be idiosyncratic or intrinsic. Idiosyncratic DIKI is unpredictable and it often stems from a T cell response against the drug triggered by haptens presented by the human leukocyte antigen (HLA) molecules to the T cells (e.g., abacavir). Intrinsic DIKI is the result of a dose-dependent toxic effect of a drug and/or the metabolites thereof, often at the expense of mitochondria, which is followed by cell death and renal dysfunction (Mehta et al., 2015).

Tubular cells are highly enriched in mitochondria to meet the high ATP demand to sustain the activity of the ATPase pumps and the transmembrane electrochemical gradients that drive the secretion and reabsorption of solutes (Soltoff, 1986). Mitochondrial ATP production is strictly oxygen-dependent as the oxidation reaction terminates with the reduction of an oxygen molecule to a water molecule in the final step of the mitochondrial electron transfer. Cytosolic ATP production is instead oxygen-independent and it is exclusively derived from the oxidation of carbohydrates that enter the anaerobic glycolytic pathway. Due to the decreasing corticomedullary oxygen gradient, energy metabolism is quite heterogeneous throughout the tubule. The loop of Henle, which lies in the medullary tract, relies primarily on anaerobic glycolysis, whereas the proximal and distal tubular cells, which resides in the cortex, are enriched in mitochondria and produce ATP primarily from the β-oxidation of fatty acids in the mitochondrial matrix (Clark and Parikh, 2020). Besides being the power plant of the proximal and distal tubular cells, mitochondria are also the hub of reactive oxygen species (ROS) production, which are harmless at low concentrations, but toxic to the mitochondria and the cell at high concentrations (Soltoff, 1986). The proximal segment of the tubule, which is the site of the secretion and the reabsorption of roughly 80% of the glomerular filtrate, is the most enriched in mitochondria (Soltoff, 1986). Another distinctive feature of the proximal tubular cells is the relatively high expression level of a series of polyspecific solute transporters, which can be occasionally hijacked to transport drugs from the peritubular blood into the urine or vice versa (Table 1; Fisel et al., 2014). Transport proteins participating in tubular drug secretion and reabsorption are solute carriers (SLCs) and ATP binding cassette (ABC) pumps. The SLCs include organic anion transporters (OATs), organic cation transporters (OCTs), organic anion transporting polypeptides (OATPs), multidrug and toxic compound extrusion (MATE) proteins, and a variety of amino acids and vitamin transporters, such as the folate carriers reduced folate carrier (RFC) and the proton-coupled folate transporter (PCFT). These transporters are passive or secondary active transporters that facilitate the movement of substrates toward or against their electrochemical gradient (Verrey et al., 2009; Zhao et al., 2011; Hagenbuch and Stieger, 2013; Koepsell, 2013; Motohashi and Inui, 2013; Samodelov et al., 2019). The ABC pumps are active transporters with bifunctional features: ATP hydrolysis and transport of the substrate. In the kidneys, the multidrug resistance proteins (MRPs) 2 and 4 and the breast cancer resistance protein (BCRP) appear to be particularly relevant in the elimination of drugs (Leier et al., 2000; Smeets et al., 2004; Huls et al., 2008).

**Table 1:** List of drugs associated with acute kidney injury and mitochondrial damage.

Class	Name	Transporter	Target	References
Antibiotic	Colistin	Megalin, PEPT2, and OCTN2	Mitochondrial membrane AMID^*^	(Teuber and Bader, 1976; Suzuki et al., 2013; Deris et al., 2014; Lu et al., 2016; Visentin et al., 2017)
	Doxycycline	OATs	Mitochondrial ribosome	(Clark-Walker and Linnane, 1966; Babu et al., 2002; Houtkooper et al. 2013; Moullan et al., 2015)
	Gentamicin	Megalin and OCT2	Mitochondrial ribosome Complex II	(Prezant et al., 1993; Moestrup et al., 1995; Qian and Guan, 2009; Gai et al., 2016; O’reilly et al., 2019)
	Vancomycin	OCTs	Complex I	(Sokol, 1991; Arimura et al., 2012; Fujiwara et al., 2012; Sakamoto et al., 2017)
Anticancer	Cisplatin	OCTs	Mitochondrial DNA VDAC	(Yang et al., 2006; Garrido et al., 2008; Filipski et al., 2009; Ciarimboli et al., 2010)
	Gemcitabine	CNTs and ENTs	DNA polimerase γ	(Mackey et al., 1998; Fowler et al., 2008)
	Ifosfamide	OCT2	Complex I	(Nissim et al., 2006; Knouzy et al., 2010; Ciarimboli et al., 2011)
	Methotrexate	FRα, RFC, PCFT, OATs, and OATPs	Mitochondrial TS	(Hoar and Dimnik, 1985; Badagnani et al., 2006; Zhao et al., 2011; Visentin et al., 2012)
	Nitrosourea derivatives	OCTs	Mitochondrial DNA	(Wunderlich et al., 1970; Pettepher et al., 1991; Chen et al., 2001).
Antifungal	Amphotericin B	Unknown	Mitochondrial membrane	(Shekhova et al., 2017)
Antiviral	Zalcitabine, Didanosine, Stavudine, Zidovudine	CNTs and ENTs	DNA polimerase γ	(Birkus et al., 2002; Pinti et al., 2006)
Others	Aristolochic acid	OATs	ANT	(Qi et al., 2007; Bakhiya et al., 2009; Xue et al., 2011)
	Carboxy-atractyloside	Unknown	ANT	(Klingenberg, 2008)

AMID, apoptosis-inducing factor-homologous mitochondrion associated inducer of death; ANT, adenine nucleotide translocase; CNT, concentrative nucleoside transporter; ENT, equilibrative nucleoside transporter; FR, folate receptor; OAT, organic anion transporter; OATPs, organic anion transporting polypeptide; OCT, organic cation transporter; PCFT, proton-coupled folate transporter; RFC, reduced folate carrier; TS, thymidylate synthase; VDAC, voltage-dependent anion channel.

* Based on phylogenetic analysis.

The high expression level of these polyspecific transporters on the membranes of proximal tubular cells and their unique mitochondrial enrichment set the conditions for the “perfect storm,” resulting in exposure of mitochondria to high concentration of drugs, massive ATP depletion, intolerable oxidative stress, and proximal tubular cell death. The resulting kidney injury usually manifests as Fanconi’s syndrome, a general malabsorption of electrolytes and substances due to proximal tubule malfunctioning that can progress to renal insufficiency (Heidari, 2019). As part of the article collection “Mitochondria in Renal Health and Disease,” this article provides an overview of the molecular mechanisms underlying drug-induced mitochondrial damage in DIKI. Sources for this review were obtained through extensive literature searches of publications browsing PubMed. Only papers published in the English language were considered.

## Mitochondrial Membranes

The mitochondrion is made of two membranes, known as the outer mitochondrial membrane and the inner mitochondrial membrane. The outer membrane is smooth and relatively permeable to solutes smaller than 5 kDa. It has a low protein density and is considered important for the physical and signaling interaction between the mitochondrion and the other subcellular compartments. The inner mitochondrial membrane is relatively impermeable to solutes and has a high protein:lipid ratio allowed by its deeply convoluted shape (Giacomello et al., 2020). The other distinctive feature of the inner membrane is the presence of cardiolipin, a lipid that is also found in bacterial membranes. Cardiolipin appears to be essential for the mitochondrial respiration and pathological changes in cardiolipin amount or species composition have been shown to alter mitochondrial respiration, boosting the production of reactive oxygen species (Horvath and Daum, 2013; Dudek, 2017).

Experiments in which the polymyxin-resistant *Acholeplasma laidlawii B* bacteria, lacking the cell wall, were fused with vesicles loaded with different lipid species, revealed that cardiolipin and phosphatidylglycerol might represent the polymyxin receptor molecules in the prokaryotic membranes (Teuber and Bader, 1976). Polymyxins are cyclic heptapeptide with an acylated tripeptide side chain, which disrupts the cell membrane integrity of the Gram-negative bacteria (Deris et al., 2014; Yu et al., 2015). Polymyxins are reabsorbed at the level of the proximal tubule by endocytosis and facilitated diffusion. The endocytic uptake seems to involve the glycoprotein megalin (Suzuki et al., 2013). In contrast, the human peptide transporter 2 (PEPT2) and the carnitine/organic cation transporter novel 2 (OCTN2), both highly expressed on the brush border membrane of proximal tubular cells, have been shown to facilitate the transport of polymyxins in overexpressing *in vitro* systems (Horvath and Daum, 2013; Lu et al., 2016; Visentin et al., 2017). Though destabilization of the plasma membrane is considered the primary mechanism of polymyxin toxicity, there are growing evidence that polymyxins induce also mitochondrial damage. The target of polymyxins in mitochondria is not known; however, it might be speculated that the interaction with cardiolipin might trigger mitochondrial membrane depolarization and apoptosis observed in proximal tubular cells (Dai et al., 2013, 2014).

Amphotericin B is an antifungal agent widely administered against invasive fungal infections; however, its clinical application is limited by the frequent onset of nephrotoxicity (Heidari, 2019). The mechanism by which amphotericin B accumulates in the proximal tubular cells is unclear. *In vitro* experiments showed that amphotericin B is a strong inhibitor of the human OCT2 but not its substrate (Trejtnar et al., 2014). Experiments in *Aspergillus fumigatus* showed that amphotericin B binds to the membrane lipids of the mitochondrial membranes, inducing loss of integrity, ROS production, oxidative stress, and lipid peroxidation (Shekhova et al., 2017). Notably, amphotericin B-induced oxidative stress can be markedly reduced by inhibiting the complex I of the electron transport chain, the major site of ROS formation (Mesa-Arango et al., 2014; Shekhova et al., 2017).

## Mitochondrial DNA Replication, Transcription, and Translation

Mitochondrial DNA is double stranded like the nuclear DNA, but circular instead of linear. The mitochondrial genome encodes exclusively for two ribosomal RNAs (rRNAs), 22 transfer RNAs (tRNAs), and the messenger RNA (mRNA) of 13 protein subunits that are assembled with nuclear-encoded proteins to form the four enzymatic complexes constituting the mitochondrial electron transport chain. Mitochondrial genes are transcribed in a polycistronic manner, whereas nuclear genes are usually transcribed as individual mRNAs. Mitoribosomes, mitochondrial tRNAs, and mRNAs form the translation apparatus; however, the translation process is completely dependent on various nuclear-encoded regulatory proteins (D’souza and Minczuk, 2018). Inhibition of mitochondrial DNA replication and/or protein synthesis, described for multiple classes of drugs associated with acute kidney injury (Table 1), results in the reduction of the steady-state level of mitochondrion-encoded proteins, a phenomenon known as mitonuclear protein imbalance, which causes reduced cellular respiration and altered mitochondria dynamics (Houtkooper et al., 2013).

### DNA Replication

The replication of the mitochondrial genome relies on the expression and activity of the DNA polymerase γ, which is encoded by the nuclear gene *POLG*. The DNA polymerase γ has two domains: a catalytic one with polymerase activity and an exonuclease one that corrects misincorporations during DNA replication (Kaguni, 2004). Direct inhibition of the DNA polymerase γ activity is considered the mechanism underlying acyclic nucleotide analogue-induced acute kidney injury (Leowattana, 2019). Acyclic nucleotide analogues are widely used to prevent the replication of viruses, such as hepatitis B virus (e.g., lamivudine), hepatitis C virus (e.g., ribavirin), herpes simplex (e.g., acyclovir), and human immunodeficiency virus (HIV; e.g., zidovudine). The uptake and accumulation of acyclic nucleotide analogues are mainly mediated by concentrative nucleoside transporters (CNTs) and equilibrative nucleoside transporters (ENTs) transporters, which are expressed throughout the body and at relatively high levels in the proximal tubular cells. Once inside the cell, acyclic nucleotide analogues must be phosphorylated through three consecutive phosphorylation steps to be able to interact with the viral DNA polymerase. Of crucial importance is the first phosphorylation step, which is the only one ensured by the viral machinery, whereas the other two reactions are mediated by the host kinases. In the triphosphate form, the drugs interact, as competitive inhibitors or alternate substrates, with the dNTPs, and if they are incorporated into the DNA strand, they terminate its extension (De Clercq, 2003). Such interaction is not specific for the viral polymerase. Experiments in different mammalian cells, including human primary renal proximal tubule epithelial cells, with zalcitabine, didanosine, stavudine, and zidovudine suggest that acyclic nucleotide analogues directly interfere with mitochondrial DNA replication catalyzed by the DNA polymerase γ. The ratio between mitochondrial DNA and chromosomal DNA, and the expression of mitochondrial proteins is markedly reduced by the exposure to acyclic nucleotide analogues, leading to mitonuclear protein imbalance. In line with a reduced oxygen-dependent ATP production, the treatment with acyclic nucleotide analogues has been shown to induce the production of lactate *in vitro*, likely as a result of a glycolytic boost (Birkus et al., 2002; Pinti et al., 2006). The inhibition of the DNA polymerase γ by acyclic nucleotide analogues can occur through four different mechanisms: (i) replication is halted, (ii) competitive inhibition with natural nucleotides, (iii) increased error rate in the mitochondrial DNA replication inhibiting the exonucleolytic proofreading function of polymerase γ, and (iv) decreased exonuclease activity (Lewis et al., 2003). It has been found that the extension of the nucleotide moiety through a stable phosphate-carbon bond (phosphonate) results in a markedly reduced mitochondrial and renal toxicity (Verhelst et al., 2002; Izzedine et al., 2005; Fernandez-Fernandez et al., 2011). This chemical modification leads to a profound alteration of the cellular pharmacology and toxicology of acyclic nucleotides: (i) due to their negative charge, the uptake of acyclic nucleotide phosphonates in the kidney cortex is primarily mediated by the organic anion transporter 1 (OAT1) and not by CTNs and ENTs, (ii) acyclic nucleotide phosphonates are direct substrates of the cellular kinases, bypassing the virus-dependent phosphorylation, being effective in the treatment of kinase deficient viruses, (iii) in contrast to the phosphate group (attached through a P-O-C bond), the phosphonate group cannot be cleaved off by cellular hydrolases, facilitating the cellular retention of the drug (Cihlar et al., 1999; Ho et al., 2000; Uwai et al., 2007), and (iv) acyclic nucleotide phosphonates, unlike acyclic nucleotide analogues, are weak inhibitors of the DNA polymerase γ. Though swollen and dysmorphic mitochondria and depletion of mitochondrial DNA have been described in patients who experienced nephrotoxicity upon treatment with acyclic nucleotide phosphonates (Tanji et al., 2001; Saumoy et al., 2004), *in vitro* experiments demonstrated that acyclic nucleotide phosphonates do not interfere with mitochondrial DNA synthesis and do not induce mitochondrial toxicity and renal toxicity in mammalian cells (Birkus et al., 2002). Likely, the mitochondrial toxicity observed in those patients was not related to the exposure to the acyclic nucleotide phosphonates but rather to the co-treatment with acyclic nucleotide analogues and hydroxyurea, both highly nephrotoxic (Tanji et al., 2001; Saumoy et al., 2004; Singh and Xu, 2016). The inhibition of the DNA polymerase γ has been also reported for gemcitabine, a pyrimidine nucleoside analog employed in the treatment of a number of cancers. Though peripheral neuropathy and hematological dysfunction are the most common side effects of this drug, several cases of kidney injury have been reported (Heidari, 2019). Like acyclic nucleotide analogues, gemcitabine is taken up by CNTs and ENTs (Mackey et al., 1998) and accumulates in the cytoplasm and in the mitochondria, inducing swelling of the mitochondria and function alteration (Heidari, 2019). Pre-steady state kinetic experiments showed that gemcitabine directly inhibits the activity of the human DNA polymerase γ (Fowler et al., 2008).

The inhibition of mitochondrial DNA replication can also occur as a consequence of DNA covalent modifications, which are a probable mechanism underlying alkylating agent-induced mitochondrial damage such as platinum and nitrosourea derivatives. Cisplatin, used as first-line treatment of various types of cancer, is a first-generation platinum-based drug and the most nephrotoxic among the platinum derivatives (Miller et al., 2010). The uptake of cisplatin in the proximal tubular cells is primarily mediated by the OCT2, which is highly expressed on the basolateral membrane, thus it is likely to mediate the entry step of cisplatin tubular secretion. After treatment with cisplatin, the double knockout mouse Oct1/2^−/−^ displayed a milder nephrotoxicity as compared with the wild-type animal. Moreover, patients carrying the nonsynonymous single-nucleotide polymorphism (SNP) in the OCT2 gene *SLC22A2* (rs316019), causing a reduction in the transport activity, have been associated with a reduced risk of cisplatin-induced nephrotoxicity (Filipski et al., 2009; Ciarimboli et al., 2010). There is a large body of evidence that the toxic effects of cisplatin are primarily the result of inter‐ and intra-strand covalent adduct formation between platinum complexes and DNA. Interestingly, dissociation-enhanced lanthanide fluoroimmunoassay studies performed in Chinese hamster ovary (CHO) cells demonstrated that the rate of cisplatin-DNA adduct incorporation was much higher in mitochondrial DNA than in genomic DNA (Olivero et al., 1995), which leads to inhibition of the DNA amplification by polymerase chain reaction (PCR) and impairment of mitochondrial DNA stability and RNA synthesis (Garrido et al., 2008). Using a panel of cancer cell lines, it has been observed that, unlike the complex I inhibitor rotenone and the uncoupler carbonyl cyanide 3-chlorophenylhydrazone (CCCP), which instantaneously alter cellular oxygen consumption, cisplatin inhibited mitochondrial respiration only after hours of exposure, a timeframe that is consistent with a mitonuclear protein imbalance-dependent effect (Gerschenson et al., 2001; Alborzinia et al., 2011). Noteworthy, it has been shown that there is a positive correlation between the cellular density of mitochondria and cisplatin-induced toxicity (Qian et al., 2005), an observation that provides another explanation for the sensitivity of proximal tubular cells to cisplatin. Damage at the level of the mitochondrial DNA is likely to be also the primary mechanism of nephrotoxicity induced by antineoplastic nitrosourea derivatives (Perry and Weiss, 1982; Narins et al., 1990). After incubation of isolated mitochondria or cell nuclei with radiolabeled methyl-N-nitrosourea, the incorporation into the mitochondrial DNA was 3–7 times that of the nuclear DNA (Wunderlich et al., 1970). Likewise, after intraperitoneal injection in rats of nitrosourea derivatives, the extent of the DNA damage was much higher in the mitochondria than in the nuclei (Wunderlich et al., 1970; Pettepher et al., 1991). The mechanism of accumulation of nitrosourea derivatives in the proximal tubular cells is not fully understood, though association studies have indicated that the expression of organic cation transporters correlated with the cytotoxic effect of nitrosourea derivatives *in vitro* (Noe et al., 1996; Chen et al., 2001). The mitochondrial selectivity of alkylating agents might be explained by a higher accumulation of these drugs in the mitochondria as compared with the nuclei and the lack of histones, which could make the mitochondrial DNA more vulnerable than the genomic DNA to the attack of these alkylating agents (Wunderlich et al., 1970).

Mitochondrial mutations have been observed upon treatment with methotrexate *in vitro*. Methotrexate belongs to the antifolates, the first class of antimetabolites to enter the clinics in the 1950s. Methotrexate inhibits the dihydrofolate reductase, rapidly depleting the cells of tetrahydrofolate derivatives that are key cofactors for the synthesis of thymidylate and purines, the building blocks of DNA and RNA synthesis. The uptake of methotrexate in the proximal tubular cells is mediated by the glycosylphosphatidylinositol (GPI) anchored high-affinity folate receptor α (FRα), by the specific folate transporters RFC and PCFT, and by OATs and OATPs (Badagnani et al., 2006; Zhao et al., 2011; Visentin et al., 2012; Samodelov et al., 2019). The etiology of methotrexate-induced renal dysfunction is believed to be primarily the consequence of the precipitation of the drug in the renal tubules because of its poor solubility at acidic pH (Jacobs et al., 1976; Smeland et al., 1996). Yet, it has been proposed that methotrexate can exert its toxic effect by accumulating in the mitochondrial matrix *via* the mitochondrial folate transporter (MFT; Titus and Moran, 2000). *In vitro* experiments indicated that the mutagenesis induced by methotrexate in non-proliferating cells seems to occur solely at the level of the mitochondrial DNA, sparing the nuclear genome. Because similar results were obtained with 5-fluorodeoxyuridine, the deoxyribonucleoside derivative of 5-fluorouracil, another thymidylate synthase inhibitor widely used in cancer treatment, it is likely that the alteration in the mitochondrial thymidylate pool underlies methotrexate-induced mitochondrial DNA mutations (Hoar and Dimnik, 1985). Notably, though 5-fluorouracil has been shown to cause nephrotoxicity in rats, it is not nephrotoxic in patients at clinically relevant doses (Rashid et al., 2014). The basis for the mitochondrial DNA selectivity of these drugs may be due to the difference in the origin of the thymidylate pool that are incorporated in the mitochondrial DNA and in the nuclear DNA. The *de novo* synthesis of thymidine triphosphate relies on the thymidylate synthase enzyme, which catalyzes the methylation of deoxyuridine monophosphate to thymidine monophosphate. Alternatively, thymidine monophosphate can also synthesize from a salvage pathway catalyzed by the cytosolic thymidine kinase 1. The expression level of thymidylate synthase and thymidine kinase 1 is cell cycle-dependent; thus, it is found in trivial amount in quiescent cells (Ke and Chang, 2004; Le Francois et al., 2007). Mitochondrial thymidine kinase 2 plays a pivotal role in thymidine triphosphate synthesis for mitochondrial DNA replication, which occurs also in non-dividing cells. Because of their differential expression in quiescent cells, such as proximal tubular cells, the inhibition of mitochondrial thymidine kinase 2, rather than thymidylate synthase and thymidine kinase 1, is likely to contribute to methotrexate-induced renal toxicity.

### Protein Synthesis

Eukaryotic cells arose from the endosymbiosis of a α-proteobacterium with a host cell with no mitochondria. Mitochondria still harbor remnants of the ancestral bacterial genome and have retained their own transcriptional and translational machinery (Gray et al., 1999). Thus, it is not surprising that mitochondrial protein synthesis is often the target of antibiotics initially developed to inhibit bacterial protein synthesis.

Aminoglycosides are potent inhibitors of the bacterial protein synthesis, whose clinical use is limited by the rapid onset of nephrotoxicity (and ototoxicity). The tropism of aminoglycosides for the kidneys (and the inner ear) is likely due to the preferential accumulation of this class of antibiotics in these tissues mediated by the low-density lipoprotein receptor-related protein megalin (Moestrup et al., 1995) and the OCT2 (Gai et al., 2016), both highly expressed in these tissues. The relevance of OCT2 in aminoglycoside-induced kidney injury is supported by the clinical observation that male and obese individuals, both characterized by a high expression level of OCT2, carry a higher risk than women or lean individuals of developing toxicity during aminoglycoside therapy (Corcoran et al., 1988; Urakami et al., 2000). Aminoglycosides interfere with bacterial protein synthesis by binding to the internal loop in helix44 of the 16S ribosomal RNA in the 30S subunit of bacterial ribosomes and to the terminal hairpin of helix69 of the bacterial large ribosomal subunit (Borovinskaya et al., 2007), which plays a critical role in ribosomal recycling and translocation (Woodcock et al., 1991; Wang et al., 2012). The aminoglycosides also cause a miscoding of mRNA codons, and, consequently, incorrect amino acids are incorporated into growing peptide chains producing faulty bacterial proteins (Davis, 1987). However, aminoglycosides also bind with lower affinity to mitochondrial ribosomes, exerting a toxic effect also on host cells in the event of high intracellular drug concentrations. By studying the mitochondrial 12S rRNA A1555G mutation, a well-known susceptibility factor in aminoglycoside-induced hearing loss, it was observed that the G1555 variant has higher affinity for aminoglycosides, directly demonstrating that human mitochondrial 12S rRNA is a target of aminoglycosides (Prezant et al., 1993; Qian and Guan, 2009). Moreover, the aminoglycosides exhibit similar binding affinities to the mitochondrial helix69. The interaction enhances the conformational stability of mitochondrial helix69 and increases the structural stability of the rRNA, hindering ribosomal recycling (Hong et al., 2015).

Tetracycline antibiotics are used for the treatment of a broad range of microorganism infections, including those induced by Gram-positive and Gram-negative bacteria. Though they have been primarily associated with liver-induced steatosis, tetracyclines can also induce nephrotoxicity (Lew and French, 1966; Phillips et al., 1974). In the 1960s, several case reports linked the onset of an acquired and usually reversible Fanconi’s syndrome with the use of tetracyclines. The authors ascribed the toxic effect to the degradation product epi-anhydrotetracycline, which induced proximal tubular necrosis in rats and dogs (Benitz and Diermeier, 1964; Tapp et al., 1965). Uptake of tetracyclines in the proximal tubular cells is likely mediated by the OAT1, OAT2, OAT3, and OAT4, which are highly expressed on the brush border and the basolateral membranes of the proximal tubular cells (Babu et al., 2002). By exploiting their intrinsic fluorescence, it was demonstrated that tetracyclines rapidly accumulate in the mitochondria (Du Buy and Showacre, 1961). Like aminoglycosides, tetracyclines inhibit bacterial protein synthesis by binding to the ribosomal 30S subunit, preventing the association of aminoacyl-tRNA with the bacterial ribosome (Chopra and Roberts, 2001). Tetracyclines inhibit the translation of proteins encoded by the mitochondrial DNA, but not by the nuclear DNA, leading to mitonuclear protein imbalance (Clark-Walker and Linnane, 1966; Houtkooper et al., 2013; Moullan et al., 2015). Indeed, the gene expression profile of the human bladder cancer cell line RT112 exposed to doxycycline indicated a global repression of the mitochondrial protein synthesis and function (Moullan et al., 2015). Also, mitochondria isolated from the liver of mice treated with doxycycline were characterized by decreased mitochondrial respiration and ATP content, and repression of several mitochondrial genes (Moullan et al., 2015). The extensive experimental evidence on the impact of tetracyclines on mitochondria not only shed light on the molecular mechanisms of tetracycline-induced toxicity but also raised awareness within the scientific community on the use of these antibiotics for inducible gene expression in the Tet-ON/Tet-OFF system. The mitochondrial dysregulation associated with the use of these antibiotics may be a relevant pitfall in the study of mitochondrial-related disorders (Chatzispyrou et al., 2015; Moullan et al., 2015; Luger et al., 2018).

## Electron Transport Chain

Reducing equivalents generated from the metabolism of glucose, lipids and amino acids flow into the tricarboxylic acid cycle and the electron transport chain to ultimately produce ATP. The tricarboxylic acid cycle is characterized by eight enzymatic steps, which generate flavin adenine dinucleotide (FADH_2_) and nicotinamide adenine dinucleotide (NADH) from FAD and NAD^+^. These reduction-oxidation reactions feeds the electron transport chain, a series of multi-subunit proteins, prosthetic groups, and cofactors embedded in the inner mitochondrial membrane. A second series of reduction-oxidation reactions catalyzed by the electron transport chain generates the inward proton gradient required to drive the synthesis of ATP mediated by the F_0_F_1_-ATP synthase. Five complexes constitute the electron transport chain with complex V being F_0_F_1_-ATP synthase. Especially, complex I and complex II appear to be the target of a number of widely prescribed drugs (Alberts et al., 2002).

### NADH:Ubiquinone Oxidoreductase – Complex I

Complex I oxidizes NADH, transferring two electrons to ubiquinone (Coenzyme Q, CoQ), a lipophilic electron carrier, and four protons into the intermembrane space. Complex I, together with complex II, is the primary source of ROS production in the cell. The most common complex I inhibitor is rotenone though 60 different families of compounds with inhibitory effect at the level of complex I have been identified thus far (Lenaz et al., 2006). Examples of complex I-related DIKI are the anticancer chemotherapeutic ifosfamide and the antibiotic vancomycin.

Ifosfamide is an alkylating agent used in anticancer chemotherapeutic protocols for treating several malignancies, including testicular cancer, soft tissue sarcoma, osteosarcoma, and bladder cancer. The treatment with ifosfamide but not with its structural isomer cyclophosphamide has been associated with tubular dysfunction. The uptake of ifosfamide in proximal tubular cells is mediated primarily by OCT2 and coadministration of cimetidine, another OCT2 substrate, completely prevented ifosfamide-induced toxicity in isolated mouse proximal tubules. Notably OCT2 does not transport cyclophosphamide (Ciarimboli et al., 2011). The mitochondria of the renal cortex from rats treated with ifosfamide showed a 50% decrease in State 3 respiration with complex I substrates but not with complex II substrates, suggesting that complex I inhibition might underlie ifosfamide-mitochondrial toxicity. Moreover, it has been shown that chloroacetaldehyde, an ifosfamide metabolite, inhibits complex I in rat renal cortical slices as well (Nissim et al., 2006; Knouzy et al., 2010). Co-injection with agmatine, a stimulator of fatty acid β-oxidation, sustained oxidative phosphorylation in the presence of ifosfamide. The authors suggested that agmatine might bypass the block of chloroacetaldehyde at the level of the complex I by feeding the complex II. However, it is worth to mention that agmatine is also a substrate of OCT2, hence the co-administration of agmatine might as well reduce OCT2-mediated uptake of ifosfamide in proximal tubular cells (Nissim et al., 2006; Winter et al., 2011). In patients treated with carboplatin and ifosfamide, who developed Fanconi-like syndrome, decreased expression of the complex III and IV and deletions in mitochondrial DNA were observed (Di Cataldo et al., 1999). However, this finding might be the result of the interaction of carboplatin with mitochondrial and nuclear DNA rather than that of ifosfamide with complex I.

Vancomycin is an amphoteric glycopeptide antibiotic used to treat Gram-positive bacterial infections that do not respond to other antibiotics. Though not fully characterized, vancomycin selectively accumulates in the proximal tubular cells by active tubular secretion from the peritubular blood likely mediated by organic cation transporters (Sokol, 1991; Fujiwara et al., 2012). The only known biological target of vancomycin is the bacterial cell wall. Vancomycin inhibits the biosynthesis of bacterial cell wall peptidoglycan by binding C-terminal acyl-D-alanyl-D-alanine (acyl-d-Ala-d-Ala)-containing residues in peptidoglycan precursors (Reynolds, 1989), a prokaryotic structure. Such selectivity is difficult to reconcile with any off-target effect in the proximal tubular cells, suggesting that, perhaps at very high intracellular concentrations, the vancomycin antibacterial effect might be pleiotropic and most probably less specific. Experiments with LLC-PK1 cells showed that vancomycin binds to and inhibit complex I activity, stimulating the production of superoxide, leading to peroxidation of the mitochondrial phospholipid cardiolipin and mitochondrial membrane depolarization followed by activation of the intrinsic apoptotic pathway. Carbonyl cyanide-4-(trifluoromethoxy) phenylhydrazone (FCCP), a protonophore that dissipates the proton gradient of the mitochondrial membrane, thereby inhibiting superoxide production and reducing cardiolipin peroxidation, and ameliorates vancomycin mitochondrial toxicity, suggesting that the ROS generated upon complex I inhibition promotes cardiolipin peroxidation followed by mitochondrial membrane depolarization and apoptosis (Orrenius, 2007; Arimura et al., 2012; Hanske et al., 2012; Sakamoto et al., 2017).

There is evidence that polymyxins might inhibit bacterial cellular respiration not only by interacting with the mitochondrial membrane lipids but also by binding to the complex I (Tochikubo et al., 1986; Mogi et al., 2009). The prokaryotic respiratory chain consists of three complexes that move electrons and protons between large protein complexes (Kim and Kim, 2004; Kerscher et al., 2008). Complex I consists of three inner membrane respiratory enzymes: the proton-translocating NADH-quinone (Q) oxidoreductase (NDH-1), the NADH-Q oxidoreductase (NDH-2), and the sodium-translocating NADH-Q oxidoreductase (Yagi et al., 1998). Polymyxins have been shown to inhibit the NDH-2 activity in a non-competitive manner (Deris et al., 2014). Phylogenetic comparisons clustered the human protein apoptosis-inducing factor-homologous mitochondrion associated inducer of death, AMID (AIF-M2) with the prokaryotic NDH-2 family and not in the group containing the canonic AIF proteins, mitochondrial flavoproteins with oxidoreductase activity (Marshall et al., 2005; Elguindy and Nakamaru-Ogiso, 2015; Marreiros et al., 2016).

### Succinate-Coenzyme Q Reductase – Complex II

Complex II is the succinate dehydrogenase, an enzyme also involved in the oxidation of succinate to fumarate as part of the tricarboxylic acid cycle. As part of the electron transport chain, succinate dehydrogenase, like complex I, transfers electrons from succinate to ubiquinone (Bezawork-Geleta et al., 2017). Complex II-mediated ROS production is considered key in ischemia/reperfusion injury (Ralph et al., 2011). It has been shown that the antibiotic gentamicin, the most commonly prescribed aminoglycoside, is not only an inhibitor of mitochondrial protein synthesis but can also directly interact with complex II. In cochlear sensory hair cells, gentamicin stimulates state 4 and inhibits state 3u respiratory rates by inhibiting complex II of the electron transport chain, thereby reducing the respiratory control ratio and collapsing the mitochondrial membrane potential as an uncoupler of the electron transport chain. As already known for other uncouplers, gentamicin reduced ROS production in isolated mitochondria (O’reilly et al., 2019). By contrast, intact cells and animals treated with aminoglycosides overproduce ROS (Cuzzocrea et al., 2002; Kalghatgi et al., 2013), perhaps in a mitochondrion-independent manner. In fact, it has been shown that gentamicin can also form ternary complexes with iron and lipid and catalyze ROS formation (Lesniak et al., 2005).

## Mitochondrial Permeability Transition Pore

The permeability transition represents a sudden increase of the inner mitochondrial membrane permeability to solutes with a molecular weight lower than 1.5 kDa. Short-term openings of the permeability transition pore represent physiological adjustments to regulate Ca^2+^ and ROS homeostasis, providing mitochondria with a fast mechanism to release Ca^2+^ when this reaches a harmful concentration inside the mitochondrial matrix. Conversely, long-term opening of the pore is linked to mitochondrial dysfunction because its occurrence leads to mitochondrial depolarization, cessation of ATP synthesis, Ca^2+^ release, pyridine nucleotide depletion, and inhibition of respiration. *In vitro*, long-term opening of the pore lead to matrix swelling, which, in turn, causes the mobilization of cytochrome c, the outer mitochondrial membrane rupture, and eventually the release of proapoptotic proteins such as cytochrome c. The precise molecular composition and identity of the mitochondrial permeability transition pore are still controversial. Candidate components of the pore are the adenine nucleotide translocase (ANT), the voltage dependent anion channel (VDAC), the phosphate carrier (PiC), and components of the ATP synthase (Bernardi and Di Lisa, 2015).

It has been suggested that taxane-induced and platinum-induced mitochondrial injury might be associated to a direct interaction with the permeability transition pore. Though there is a large body of evidence that therapeutic and toxic effects of cisplatin on cells is primarily a consequence of inter-strand and intra-strand covalent adduct formation with nucleic acids, it has been shown that platinum can also crosslink with proteins. Experiments in intact cells suggest that cisplatin, perhaps conjugated to glutathione, accumulates in mitochondria, rapidly impairs the oxygen consumption, and induces oxidative stress (Dzamitika et al., 2006; Garrido et al., 2008). Depending on the source, oxidative stress not only can originate from mitochondria but also from other organelles, especially endoplasmic reticulum (ER) and peroxisomes (Kaludercic et al., 2014). Co-localization experiments in Human Kidney-2 (HK-2) cells demonstrated that cisplatin-induced ROS is of mitochondrial origin (Choi et al., 2015). It has been shown that cisplatin can form crosslinks with the VDAC, facilitating mitochondrial membrane permeabilization, release of cytochrome c, and apoptosis (Yang et al., 2006). Short exposure to paclitaxel produces marked loss of renal tubules epithelial lining and damage of the brush border membranes with signs of both oncotic necrosis and apoptosis (Rabah, 2010). Paclitaxel induces an abrupt fall of the mitochondrial membrane potential and a loss of mitochondrial Ca^2+^. Because cyclosporin A, a de-sensitizer of the mitochondrial permeability transition pore, blocked paclitaxel-induced loss of mitochondrial Ca^2+^, the authors concluded that paclitaxel induced the opening of the mitochondrial permeability transition pore (Kidd et al., 2002).

The opening of the transition pore has also been associated with nephrotoxicity induced by some natural products very popular in traditional medicine. *Xanthium strumarium*, used in traditional Chinese medicine to treat nasal and sinus congestion, has been linked to several cases of poisoning with renal proximal tubular necrosis features which upon *X. strumarium* ingestion have been reported in the literature (Turgut et al., 2005). The seeds of this plant are enriched in carboxyatractyloside, a well-characterized inhibitor of the ANT. The binding of carboxyatractyloside to the ANT triggers the opening of the mitochondrial transition pore (Obatomi et al., 1998; Klingenberg, 2008). In 1992, a high prevalence of kidney disease in female patients ingesting slimming pills raised attention to the nephrotoxicity of aristolochic acids. Ever since, aristolochic acids are considered a group of toxins that can cause end-stage renal failure associated with urothelial carcinomas (Han et al., 2019). Aristolochic acid is transported into the proximal tubular cells by the OATs (Bakhiya et al., 2009; Xue et al., 2011). Studies in HK-2 cells showed that aristolochic acid exposure caused ATP depletion, mitochondrial membrane depolarization, cytochrome c release, and increase of caspase 3 activity. These toxic effects were attenuated by cyclosporin A, a known “desensitizer” of the opening of the pore (Bernardi, 1996). Experiments in isolated mitochondria showed that aristolochic acid inhibited the activity of the mitochondrial ANT (Qi et al., 2007).

## Protective Strategies

In principle, there are two approaches to protect the kidneys from drug toxicity. The first approach is reducing the intracellular accumulation of the drug by selectively inhibiting its uptake. A number of works have focused on the pharmacological inhibition of OCT2. It has been shown that the inhibition of OCT2 activity with cimetidine or agmatine, two OCT2 substrates, prevents nephrotoxicity induced by drugs that are OCT2 substrates such as cisplatin and ifosfamide (Nissim et al., 2006; Ciarimboli et al., 2010, 2011). It remains unclear how metformin protects from gentamicin-induced kidney injury. Metformin, a well-known OCT2 substrate, prevents gentamicin-induced nephropathy in rats (Morales et al., 2010; Nasri, 2012). Because at that time a role for OCT2 in gentamicin transport was not considered, the effect of metformin on the renal uptake of gentamicin was not explored. Later on, it was shown that gentamicin is an OCT2 substrate as well, and that gentamicin uptake in primary cultured proximal tubular cells was suppressed by co-incubation with metformin (Gai et al., 2016). To sort out what is the prevalent protective mechanism exerted by metformin, experiments with other mitotoxic drugs that are not substrates of OCT2 and experiments in isolated mitochondria are required. The other protective approach that attracted several research groups is promoting the anti-oxidant activity of the cell, thereby coping better with the high level of oxidative stress subsequent to the mitochondrial injury. There is a plethora of *in vitro* and animal studies showing the nephroprotective effect of different types of antioxidant supplementation in DIKI, yet the subsequent human studies were not just as much practical (Heidari, 2019). Ascorbic acid, a potent reducing agent and radical scavenger, was effective against kidney injury induced by several drugs causing mitochondrial damage, including gentamicin, colistin, and cisplatin (Niki, 1991; May et al., 1995; Antunes et al., 2000; Sharma and Mongan, 2001; Yousef et al., 2012; Moreira et al., 2014). In a small clinical trial, ascorbic acid failed to protect the kidneys of patients taking colistin (Sirijatuphat et al., 2015). Supplementation with N-acetylcysteine, a sulfhydryl-donor with well-known antioxidant properties, hindered the glomerular filtration deterioration in rats treated with amphotericin B (Feldman et al., 2005). Conversely, oral N-acetylcysteine administration to patients under amphotericin B treatment did not prevent the onset of the nephrotoxicity (Karimzadeh et al., 2014).

## Conclusions

Mitochondrial injury is a common event in drug-induced toxicity. The renal liability of some drugs is likely to be the result of the high mitochondrial density in the cytoplasm of tubular cells coupled to the vast array of drug transporters highly expressed on the basolateral and brush border membrane of proximal tubular cells. To improve the safety and thus the therapeutic window of these drugs, the first important step is to study in detail their cellular and molecular pharmacology. It is of particular importance to comprehensively characterize the cellular and subcellular transport and the molecular target(s) of nephrotoxic drugs. Additionally, it is essential to optimize, in humans, dose and treatment schedule of the antioxidant supplementation approaches that have been successfully tested in animals to fill in the shortest time possible, the shortcoming of safe nephroprotective strategies. Information resulting from such targeted studies could be exploited to design pharmacological strategies, which uncouple the uptake of the drug from its toxic effect at the expense of the mitochondria by selective inhibition of the drug uptake into the proximal tubular cells and/or by reducing the oxidative stress burden derived from the mitochondrial damage.

## Author Contributions

MV contributed to the conceptualization. ZG, TG, and MV contributed to the literature search. ZG, TG, and MV prepared the writing of the original draft preparation. YL and GK-U contributed to the writing-review and editing. All authors contributed to the article and approved the submitted version.

### Conflict of Interest

GK-U was employed by the company Novartis Pharma.

The remaining authors declare that the research was conducted in the absence of any commercial or financial relationships that could be construed as a potential conflict of interest.
